# Case Report: Molecular and microenvironment change upon midostaurin treatment in mast cell leukemia at single-cell level

**DOI:** 10.3389/fimmu.2023.1210909

**Published:** 2023-08-10

**Authors:** Meng-Ke Liu, Feng Liu, Yu-Ting Dai, Xiang-Qin Weng, Li-Li Cheng, Li-Quan Fan, Han Liu, Lu Jiang, Xiao-Jian Sun, Hai Fang, Li Wang, Wei-Li Zhao

**Affiliations:** Shanghai Institute of Hematology, State Key Laboratory of Medical Genomics, National Research Center for Translational Medicine at Shanghai, Ruijin Hospital Affiliated to Shanghai Jiao Tong University School of Medicine, Shanghai, China

**Keywords:** mast cell leukemia, midostaurin, molecular changes, single-cell RNA sequencing, tumor micoenvironment

## Abstract

Mast cell leukemia is a rare and aggressive disease, predominantly with *KIT* D816V mutation. With poor response to conventional poly-chemotherapy, mast cell leukemia responded to the midostaurin treatment with a 50% overall response rate (ORR), but complete remission rate is approximately 0%. Therefore, the potential mechanisms of midostaurin resistance and the exact impacts of midostaurin on both gene expression profile and mast cell leukemia microenvironment *in vivo* are essential for design tailored combination therapy targeting both the tumor cells and the tumor microenvironment. Here we report a 59-year-old male mast cell leukemia patient with *KIT* F522C mutation treated with midostaurin. Single-cell sequencing of peripheral blood and whole exome sequencing (WES) of bone marrow were performed before and 10 months after midostaurin treatment. In accordance with the clinical response, compared to the pretreatment aberration, the decline of mast cells and increase of T-, NK, B-cells in peripheral blood, and the decrease of the *KIT* F522C mutation burden in bone marrow were observed. Meanwhile, the emergence of *RUNX1* mutation, upregulations of genes expression (*RPS27A*, *RPS6*, *UBA52*, *RACK1*) on tumor cells, and increased frequencies of T and NK cells with *TIGIT, CTLA4*, and *LAG3* expression were observed after midostaurin treatment, predicting the disease progression of this patient. As far as we know, this is the first case reporting the clinical, immunological, and molecular changes in mast cell leukemia patients before and after midostaurin treatment, illustrating the *in vivo* mechanisms of midostaurin resistance in mast cell leukemia, providing important clues to develop a sequential option to circumvent tumor progression after targeting oncogene addiction and prolong patients’ survival.

## 1 Introduction

Mast cell leukemia is a rare but aggressive form of systemic mastocytosis (SM) characterized by the expansion of neoplastic mast cells in bone marrow (BM) and peripheral blood (1). Pathogenic mutation of *KIT* D816V is found in 80% of SM; *TET2, SRSF2, ASXL1, RUNX1, JAK2*, and *RAS* are also frequently mutated and related to poor prognosis of mast cell leukemia patients (1, 2). No curative therapy is yet available for mast cell leukemia other than allogeneic hematopoietic stem cell transplantation; a median survival time in mast cell leukemia patients is less than 12 months when treated with chemotherapy or *KIT* inhibitors (2). Midostaurin is a small molecule inhibitor of multiple tyrosine kinase receptors and has been approved by the FDA and EMA as a *KIT* inhibitor in treating mast cell leukemia, with an overall remission rate of 50%, a complete remission (CR) rate of 0%, and a median progression-free survival (PFS) of 11.3 months (3). Reasons why mast cell leukemia responded to midostaurin but displayed a low CR rate and the underlying mechanisms behind relapse *in vivo* after midostaurin treatment were not clearly specified. In our study, we performed whole exome sequencing (WES) of bone marrow and single-cell RNA sequencing of peripheral blood on a mast cell leukemia patient before and after midostaurin treatment to illustrate the *in vivo* mechanism of midostaurin on mast cell leukemia.

## 2 Case presentation

**Figure 1:**
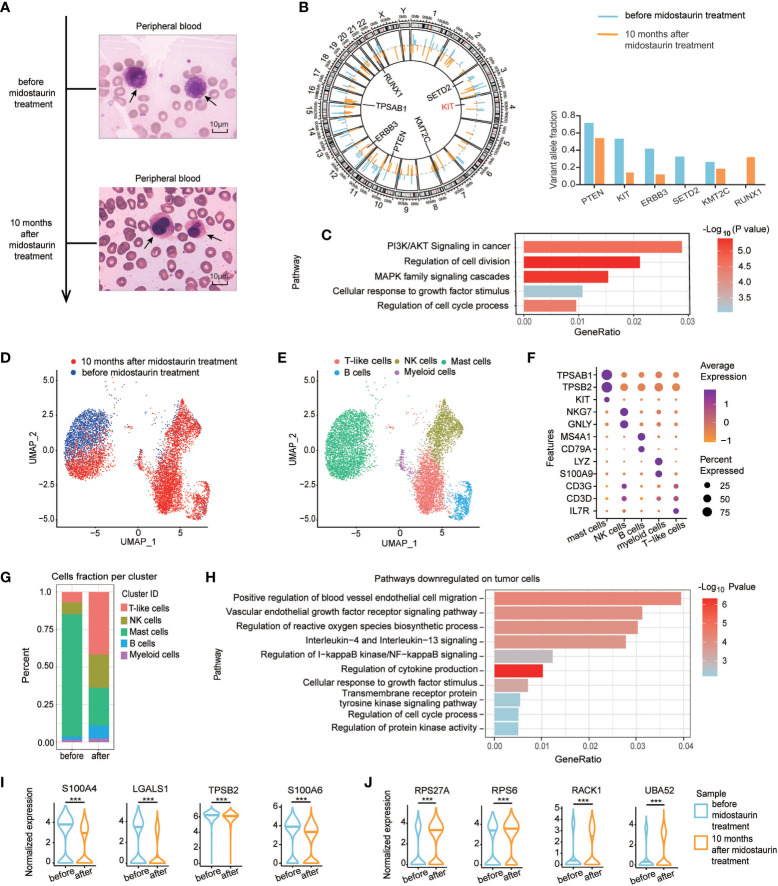
The morphological characteristics, mutation profiles, and single-cell analysis of tumor cells in the patient before and after midostaurin treatment. **(A)** Peripheral blood aspirate under Wright’s stain, demonstrating neoplastic mast cells with immature nuclear chromatin, abundant tightly packed cytoplasmic granules, high nuclear to cytoplasmic (N:C) ratio before treatment (top) and mast cells with condensed nuclear chromatin, less granules in cytoplasm and lower N:C ratio after 10 months of midostaurin treatment (bottom), bar=10μM. **(B)** Circos plot of single-nucleotide variations based on WES before and after midostaurin treatment. The outer lines colored in blue represent mutations detected before treatment. The inner lines colored in orange represent mutations detected after treatment. **(C)** Gene set enrichment analysis of genes decreased with mutation burden after treatment. **(D)** UMAP of single-cell RNA sequencing experiments of patient PBMCs before and after midostaurin treatment. Each cell represents a cell. Blue and red represent cells collected before or after 10 months of midostaurin treatment. **(E)** UMAP of single-cell RNA sequencing experiments as in **(D)** Annotated cell types are distinguished by colors. **(F)** Dot plot of cell-type-specific marker genes used to annotate the clusters in single-cell RNA sequencing experiments. **(G)** Fraction of different cell types before and after midostaurin treatment. **(H)** Pathways downregulated on tumor mast cells after midostaurin treatment. Normalized gene expression of downregulated genes **(I)**, and upregulated genes **(J)** on tumor cells after midostaurin treatment. ***, represented significant difference of P<0.001.

In our study, we reported a 59-year-old male who experienced recurrent diarrhea, severe weight loss, urticaria, anemia and weakness. He came to our hospital in June 2018 and blood tests showed white blood cells (WBCs) 18.4×10^9^/L (3.97-9.15×10^9^/L), red blood cells 1.64×10^12^/L (4.09-5.74×10^12^/L), hemoglobin 8g/dl (13.1-17.2 g/dl), platelets 142×10^9^/L (85-303×10^9^/L), and alkaline phosphatase (AKP) 196 U/L (38-126 U/L). BM examinations indicated 84% abnormal mast cells with irregular sizes and positive toluidine blue staining. Peripheral blood aspirate demonstrated neoplastic mast cells with immature nuclear chromatin, abundant tightly packed cytoplasmic granules and high nuclear to cytoplasmic (N:C) ratio (**Figure 1A**). Flow cytometric analysis was performed on BM sample before treatment to observe mast cells in circulation and used as evidence for diagnosis according to the diagnose criteria of mast cell leukemia (2). As shown in **Supplementary Figure 1**, a significant population was observed in bone marrow sample showing extremely bright expression of CD117 and positive CD45 with moderate FSC and SSC (red color). Gating on CD117 bright expression cells, the population expressed CD13, CD33, CD203c and CD2 (partially), but not lineage specific markers such as cytoplasmic MPO, CD3, CD79a or the remaining markers. The very bright expression of CD117 combining the positive of CD203c indicated the mast cell origin. The partial expression of CD2 was considered as a common feature of abnormal mast cells. We further stained CD25 and obtained negative result. WES of BM cells revealed *KIT* F522C mutation and *SETD2* mutation, but negative for *TET2*, *ASXL1*, *BCR-ABL*, *JAK2*, *FLT3*, and *NPM1* mutations (**Figure 1B**). According to the dose modification instructions of midostaurin (4), the patient was treated with 100 mg/d midostaurin due to anemia and severe fatigue, and achieved a major response 10 months after treatment, with mast cells decreasing to 24% in BM. Meanwhile, blood smears showed mast cells with condensed nuclear chromatin, less granules in cytoplasm and lower N:C ratio (**Figure 1A**). Upon midostaurin treatment, compared to the pretreatment gene mutation profiles, variant allele frequency (VAF) was also significantly decreased in genes involved with the *RAS-MAPK* pathway, such as *KIT* and *ERBB3.* However, *RUNX1* mutation, relating to poor prognosis of mast cell leukemia patients, was only detected after midostaurin treatment, which might indicate subsequent midostaurin resistance. (**Figure 1B**). Gene set enrichment analysis (GSEA) showed the top 5 downregulated pathways, including *PI3K/AKT* signaling in cancer, regulation of cell division, *MAPK* family signaling cascade, cell response to growth factor stimulus, and regulation of cell cycle process (**Figure 1C**), in which *PI3K/AKT* and *MAPK* signaling pathway are the downstream of *KIT* signaling and are associated with mast cell leukemia progression (5).

Before treatment, single-cell RNA sequencing revealed that mast cells were the major fraction (81.40%) in peripheral blood mononuclear cells (PBMC) (**Figures 1D–F**). After 10 months of midostaurin treatment, the fraction of mast cells in peripheral blood decreased significantly (from 81.40% to 25.10%), while immune cells increased, including T cells (from 6.79% to 41.67%), Natural killer (NK) cells (from 8.07% to 22.16%) and B cells (from 2.18% to 8.72%) (**Figure 1G**). The top 5 downregulated pathways on mast cells included positive regulation of blood vessel endothelial migration, vascular endothelial growth factor receptor signaling, IL-4 and IL-13 signaling, *NF-KB* signaling regulation, and cytokine production pathways regulation (**Figure 1H**). The expression level of genes such as *S100A4*, *LGALS1*, *TPSB2*, and *S100A6* were decreased. Instead, the expression level of *RPS27A, RPS6*, *UBA52*, and *RACK1*, involved in tyrosine kinase inhibitors (TKI) resistance, were increased after midostaurin treatment (**Figures 1I, J**).

**Figure 2:**
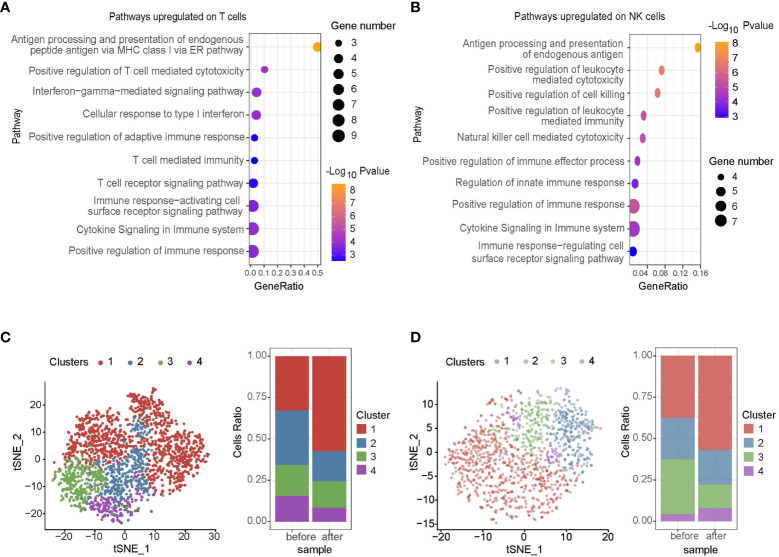
Single-cell RNA sequencing of immune cells in patient before and 10 months after midostaurin treatment. Pathways upregulated on T cells **(A)** and NK cells **(B)** after midostaurin treatment. T cell clusters and fraction of clusters **(C)**, NK cell clusters and fraction of clusters **(D)** before and after midostaurin treatment.

Regarding the impact of midostaurin on mast cell leukemia microenvironment, pathways upregulated on T cells mainly included antigen presenting, T cell cytotoxicity, interferon γ signaling, T cell receptor signaling, regulation of immune response, and T cell-mediated immunity (**Figure 2A**). Among NK cells, antigen presentation, regulation of cell killing, natural killer cells mediated cytotoxicity, leukocyte mediated cytotoxicity, regulation of cell surface receptor, immune response, and cytokine production pathways were upregulated (**Figure 2B**). However, upon further classifying T and NK cells into 4 clusters, we found that most immune checkpoints such as *TIGIT, CTLA4, LAG3*, and *HAVCR2* (also named *TIM3*) were highly expressed on cluster 1 and the cell ratios of cluster 1 in both T and NK cells were significantly increased after midostaurin treatment (P<0.05, **Figures 2C, D**). Further GSEA revealed that as compared to other clusters, the downregulated pathways on cluster 1 cells, including adaptive immune system, T cell activation, antigen processing and presentation pathways on cluster 1 of T cell (**Supplementary Figure 2A**), as well as vesicle-mediated transport, TCR, endosome to lysosome transport pathways on cluster 1 of NK cell (**Supplementary Figure 2B**), indicating the impaired functions of antigen presenting and cell cytotoxicity in these T/NK cells.

Finally, the patient had disease progression 15 months after midostaurin treatment, with a percentage of mast cells in BM reaching 77.9% in September 2019; the patient was then treated with dasatinib. Unfortunately, he had no response (without any clinical improvement) to dasatinib, but displayed severe pleural effusion. Due to the poor response to dasatinib, the patient received low dose chemotherapy with azacitidine plus homoharringtonine and arsenic trioxide, but showed no response to these treatments. Eventually, the patient died of disease progression in 2020, with severe pleural effusion and ascites, with an OS of 24 months.

## 3 Discussion

Midostaurin has significantly improved the life quality of mast cell leukemia patients with *KIT* D816V mutation. Few reports focused on its efficacy in *KIT* F522C mutated patients (3). Our patient with *KIT* F522C responded well to midostaurin as the first-line therapy and achieved 15 months of progression-free survival. *KIT* gain-of-function mutation triggers the activation of several downstream signaling, including *MAPK, JAK-STAT*, and *PI3K* pathways which regulates the proliferation, survival, and antiapoptotic function in mast cells (5). Consistent with the clinical response, we found the downregulation of *PI3K/AKT* and *MAPK* signaling pathways following midostaurin treatment.

However, during the response to midostaurin, emergent TKI resistances were observed not only in tumor cells but also in immune cells. In terms of tumor mast cells, cytokine pathways such as IL-4 and IL-13 signaling pathways, were downregulated after midostaurin treatment, thus indicating mast cells’ inactivation (6). Vascular endothelial-derived growth factor (VEGF) signaling pathway involved in activating blood vessel endothelial migration could also be attenuated through midostaurin treatment (7). These alterations in signaling pathways on tumor cells indicated that midostaurin inhibits tumor activation and metastasis. On the other hand, the upregulation of genes, such as *RPS27A, RPS6, UBA52, and RACK1* on tumor cells, involved in TKI resistance (8–11), as well as *RUNX1* mutation after midostaurin treatment, all these observations might partially explain the mast cell leukemia progression during midostaurin maintenance and afterward, the resistance to dasatinib, another TKI which has synergetic effect with midostaurin on mast cells (12). Consistent with our observation, in acute myeloid leukemia, midostaurin resistance was mainly attributed to the emergence of new mutations, including either mutation on different locus of *KIT*, or mutation on a new gene contributing to the activation of downstream signaling pathways (13). In our case, residual tumor cells with *KIT* F522C mutation after midostaurin treatment or the emergent *RUNX1* mutation-containing cells might contribute to the resistance to midostaurin. Indeed, *RUNX1* mutation was described as a risk factor in mast cell leukemia patients, and as a transcriptional regulator, *RUNX1* could activate the enhancer of *c-KIT*, trigger the transcriptional regulation of *c-KIT* to promote the proliferation of malignant cells (14). In addition to *RUNX1*, increased expression of *RPS27A, RPS6, UBA52, and RACK1* on mast cells might also contribute to midostaurin resistance. These emergent/secondary aberrations before and after midostaurin treatment thus suggested multiple mechanisms underlying the relapse of the mast cell leukemia patient. These aberrations could also be served as potential targets in further studies. For example, knockdown of *RPS27A* could arrest cell growth in TKI-resistant leukemia cell lines (15). *PI3K* pathway inhibitors could effectively dephosphorylate *RPS6* in imatinib-resistant cell lines (9); *RACK1* overexpression upregulated protein kinase C (PKC) activity. Therefore, PKC inhibitors also induced cell apoptosis in chemoresistant leukemia cell lines (16). Additionally, novel *KIT* inhibitor avapritinib, which was licensed by the FDA since 2021 and by the EMA since 2022, has distinct resistance profiles with midostaurin (17), might be an option for midostaurin-resistant patients.

Besides, tumor microenvironment had been improved, with the decrease of mast leukemia cells and increase of T and NK cells, indicating an activated antitumor condition in the patients after midostaurin treatment. In addition, single-cell RNA sequencing revealed an activation in the immune response of T cells, including upregulation of antigen presenting, T cell cytotoxicity and T cell-mediated immunity pathways (18). NK cells mediated cytotoxicity, positive regulation of cell killing, and regulation of cytokine production pathway were also activated on NK cells after treatment (18). Meanwhile, the frequencies of T and NK cells with *TIGIT, CTLA4*, and *LAG3* expression were significantly increased after midostaurin treatment, displaying the resistance mechanisms of microenvironment during the exposure to midostaurin. Consistent with our observations, TIGIT^+^ T cells or TIGIT^+^ NK cells had impaired cell function and decreased cytokine secretion, which provided a tumor-supportive microenvironment (19, 20). Because upregulation of these immune checkpoints in peripheral blood were correlated with disease relapse in leukemia (21), an increase of dysfunctional T (e.g., TIGIT^+^ T) and NK (e.g., TIGIT^+^ NK) cell clusters might contribute to the relapse of this patient. In the future, therapeutically blockading of TIGIT (e.g., anti-TIGIT antibody) might be introduced to combined therapy in mast cell leukemia to enhance the cytotoxicity of T and NK cells (22).

## 4 Conclusion

In this case, we analyzed the genetic and tumor microenvironment changes in the mast cell leukemia patient after midostaurin treatment, in order to illustrate the *in vivo* mechanisms of aquired resistance to midostaurin. Although the pathogenic gene mutation *KIT* was repressed by midostaurin when the patient achieved major response, we inferred that the arising of *RUNX1* mutation, upregulating genes expression (*RPS27A, RPS6, UBA52, RACK1*) on tumor cells and the immune suppressive tumor microenvironment with increased dysfunctional T and NK cells could contribute to the subsequent midostaurin resistance and disease progression in the patient. Our observations explain the reasons why mast cell leukemia responded to midostaurin but displayed a low CR rate (0%) and short median PFS (11.3 months) (4), indicating the underlying mechanisms behind relapse *in vivo* after midostaurin treatment. Furthermore, understanding of these mechanisms can be used to better monitor treatment response and the selection of resistant subclones, providing important clues to develop a sequential option to circumvent mast cell leukemia progression upon midostaurin treatment.

## Data availability statement

The original contributions presented in the study are included in the article/**Supplementary Material**. Further inquiries can be directed to the corresponding authors.

## Ethics statement

The studies involving human participants were reviewed and approved by Shanghai Ruijin Hospital Ethics Board. The patients/participants provided their written informed consent to participate in this study. Written informed consent was obtained from the individual(s) for the publication of any potentially identifiable images or data included in this article. Written informed consent was obtained from the participant/patient(s) for the publication of this case report.

## Author contributions

W-LZ and LW supervised the study. M-KL, X-QW, L-LC, L-QF collected clinical data and made the figures. M-KL, FL, Y-TD, HF, HL, LJ, X-JS carried out bioinformatic analysis and W-LZ, LW, M-KL drafted the manuscript. All authors contributed to the article and approved the submitted version.
