# Supplementary figures and images for: Case Report: Molecular and microenvironment change upon midostaurin treatment in mast cell leukemia at single-cell level
Source: Front Immunol. 2023 Aug 10;14:1210909. doi: 10.3389/fimmu.2023.1210909 (PMC10449247; doi:10.3389/fimmu.2023.1210909)

Liu et al. Supplementary Figure 1

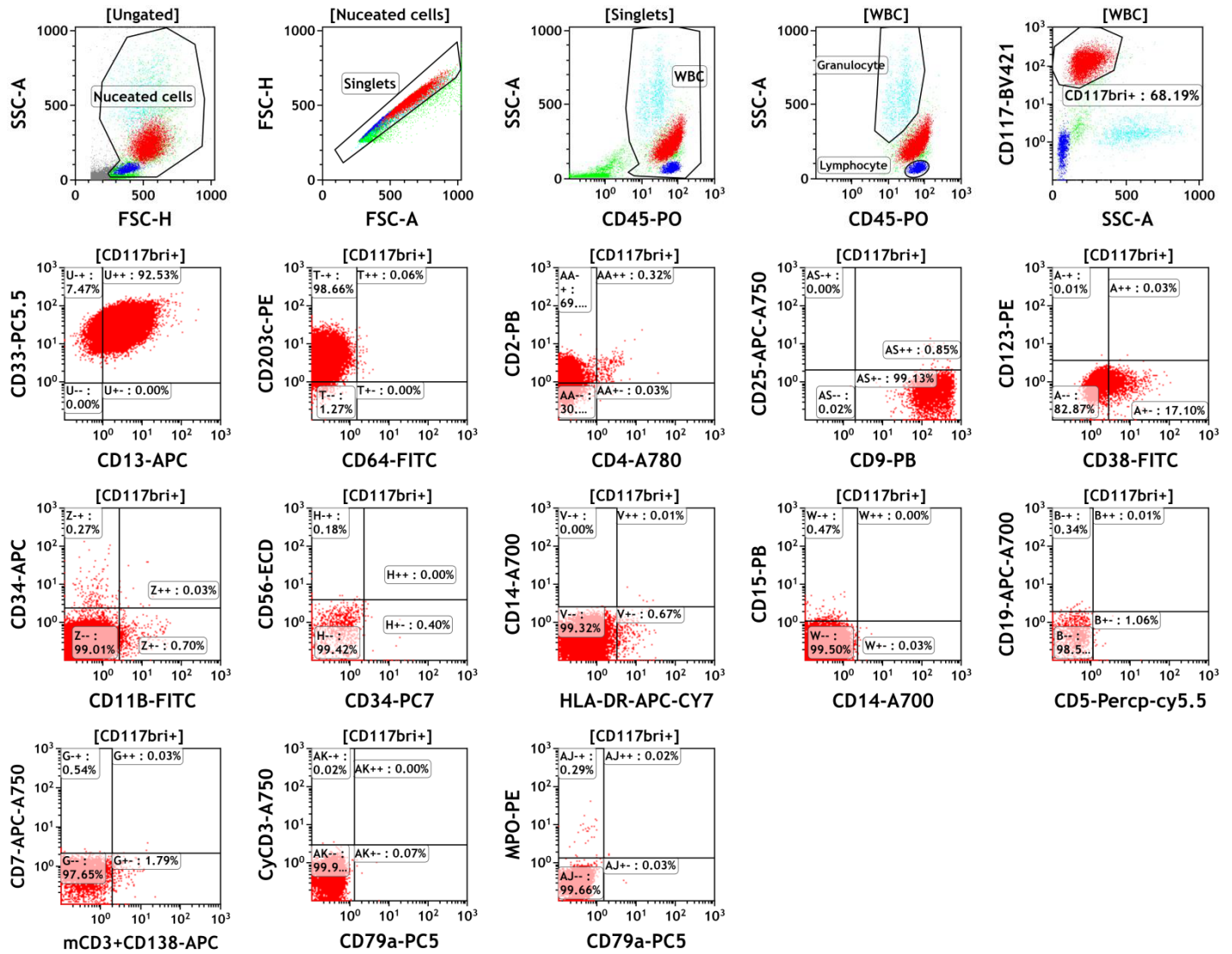

Supplement: Supplementary file 1 [file DataSheet_1.pdf]
