# Supplementary material for: Case Report: Molecular and microenvironment change upon midostaurin treatment in mast cell leukemia at single-cell level
Source: Front Immunol. 2023 Aug 10;14:1210909. doi: 10.3389/fimmu.2023.1210909 (PMC10449247; doi:10.3389/fimmu.2023.1210909)
Supplement: Supplementary file 3 [file DataSheet_3.docx]

# Supplementary Figure legends

**Supplementary Figure 1.** Gating strategy and immunophenotyping of flow cytometry before treatment. Light scatters (FSC vs. SSC and FSC-area vs. FSC-height) were used to exclude debris and doublets. The population of normal lymphocyte and granulocyte were presented by deep and light blue color. The leukemic population with extremely bright expression of CD117 was shown by red color. Gating on the CD117 bright cells, the red population displayed expression of CD13, CD33, CD203c, CD2(partially) and CD9 (brightly), but not other lineage specific markers such as cytoplasmic MPO, CD3 and CD79a. The leukemic cells were also negative for CD19, CD4, CD5, CD7, CD56, CD138, CD123, HLA-DR, CD11B, CD14, CD15, CD64, CD34 and CD25.

**Supplementary Figure 2.** Pathways downregulated on cluster 1 of T **(A)** or NK cells **(B)** compared with other T or NK clusters.
