# Supplementary material for: Case Report: Molecular and microenvironment change upon midostaurin treatment in mast cell leukemia at single-cell level
Source: Front Immunol. 2023 Aug 10;14:1210909. doi: 10.3389/fimmu.2023.1210909 (PMC10449247; doi:10.3389/fimmu.2023.1210909)
Supplement: Supplementary file 4 [file DataSheet_4.docx]

**Supplementary methods**

**Clinical characteristics**

Laboratory examination of peripheral blood was carried out with XN-9000 automatic blood analyzer and its supporting reagents. Peripheral blood and bone marrow smear were stained with Wright-Giemsa dye solution (Zhuhai Bezo Biotechnology Co., Ltd.), and morphology analysis were supported by experienced laboratory physicians.

**Flow cytometry and gating strategy**

Flow cytometric analysis was applied in immunophenotyping of bone marrow cells to observe mast cells in circulation at diagnosis. Immunophenotyping was performed by 10-color flow cytometry (NAVIOS, Beckman Coulter, Brea, California, USA). Data were analyzed with KALUZA software (Beckman Coulter, Brea, California, USA). The following antibody combinations were utilized routinely to evaluate myeloid progenitors: (1) anti-CD38-FITC / anti-CD123-PE / anti-CD5-Percp-cy5.5/ anti-CD56-BV605/ anti-CD34-PC7/anti-CD138+CD3-APC/ anti-CD19-APC-A700/ anti-CD7-APC-A750/ anti-CD117-BV421/anti-CD45-PO; (2) anti-CD64-FITC / anti-CD203c-PE / anti-CD34-PC7/ anti-CD33-PC5.5/ anti-CD117-BV421/ anti-CD13-APC / anti-CD14-A700/ anti-HLA-DR-APC-Cy7/ anti-CD15-PB /anti-CD45-PO; (3) anti-CD11B-FITC / anti-CD117-BV421/ anti-CD34-APC / anti-CD4-A780/ anti-CD2-PB / anti-CD45-PO; (4) anti-MPO-PE / anti-CD79a-PC5/anti-cyCD3-A750/ anti-CD117-BV421/ anti-CD34-APC / anti-CD45-PO. CD45, CD34 and CD117 were involved in each tube to accurately analyze antigenic expression on myeloid cells. Light scatters (FSC vs. SSC and FSC-area vs. FSC-height) were used to exclude debris and doublets initially. Then gating on the leukemic population which displayed extremely bright expression of CD117 and positive CD45 with moderate FSC and SSC. Gating on CD117 bright expression cells, the mast cell related markers CD13, CD33, CD203c, CD2, CD25 and lineage specific markers such as cytoplasmic MPO, CD3, CD79a or the remaining markers were gated separately.

**Whole exome sequencing (WES)**

Genomic DNA of the patients prior to treatment and 10 months after treatment were extracted from hair follicles (control) and bone marrow cells.

For WES, exome sequencing libraries were constructed by SureSelectXT Human All Exome V6/V6 + UTRs kit following manufacturer’s protocol, and loaded on Illumina HiSeq for 2x150 paired-end (PE) sequencing.

Bioinformatics analysis of exome sequencing raw data were applied according to the following procedures. Burrows–Wheeler Aligner (BWA) version 0.7.13-r1126 was applied to align read pairs to Human Reference Genome (version GRCh38). Samtools (version 1.3) was used to remove PCR duplications and generate chromosomal coordinate-sorted bam files. Genome Analysis Toolkit (GATK) (version 3.4) Haplotype Caller and GATK Unified Genotyper were used to call single nucleotide variations (SNVs) and indels. Annotation of SNVs and indels was applied using the UCSC Genome Browser (http://genome.ucsc.edu). Filter of SNVs and indels was based on following pipelines: 1) germline mutations detected in paired control samples were excluded; 2) mutations with low frequency (<0.1) were excluded; 3) mutations included in SNPs database and not reported in COSMIC (the Catalogue of Somatic Mutations in Cancer) version v77 were excluded.

**Drop-seq**

Single cell RNA-seq of peripheral blood mononuclear cells (PBMCs) of the patient before and 10 months after treatment was performed using Drop-seq Library Protocol version 3.1 according to Macosko et. al (1). Each library was sequenced in a flowcell lane by Illumina Xten using a 150bp paired-end sequencing kit with a custom read1 primer. After sequencing, reads were filtered and sorted by their barcodes of origin and aligned to the reference GRCh38 transcriptome using Drop-seq core computational protocol version 2.3.0 (https://github.com/broadinstitute/Drop-seq/releases). Mapped reads were quantified into UMI-filtered counts per gene. The resulted digital gene expression (DGE) matrices were imported to R programming language environment for bioinformatics analysis using Seurat (version 4.3.0). Only cells expressing at least 150 genes and cells with lees than 10% mitochondrial genes were used for downstream analysis. SCTransform was used to regress out variations of mitochondria gene expression, followed by principal component analysis using the RunPCA function. The DGE matrices containing the remaining cells of each sample were merged for graph-based clustering. Clusters were identified at the resolutions of 0.1, 0.5 and 1 using the FindClusters function and displayed on the UMAP or tSNE plot. Marker genes for each cluster were identified by FindAllMarkers function (Wilcoxon rank sum test, only.pos = TRUE, logfc.threshold = 0.2).

References:

1. Macosko EZ, Basu A, Satija R, Nemesh J, Shekhar K, Goldman M, et al. Highly Parallel Genome-wide Expression Profiling of Individual Cells Using Nanoliter Droplets. Cell. 2015;161(5):1202-14.
